# Association between patient activation, self-management behaviours and clinical outcomes in adults with type 2 diabetes: a systematic review with narrative synthesis

**DOI:** 10.1136/bmjopen-2024-095456

**Published:** 2025-05-27

**Authors:** Koghanadhacharve Thinakaran, Amy Ahern, Robert S Beckett, Sara F Shaida, Harriet M Wills, Rebecca Richards, Jack M Birch, Simon J Griffin, Julia Mueller

**Affiliations:** 1 Department of Public Health and Primary Care, University of Cambridge, Cambridge, Cambridgeshire, UK; 2 MRC Epidemiology Unit, School of Clinical Medicine, University of Cambridge, Cambridge, UK; 3 Faculty of Biology, University of Cambridge, Cambridge, Cambridgeshire, UK

**Keywords:** Patient Participation, Diabetes Mellitus, Type 2, Systematic Review, Self-Management, GENERAL MEDICINE (see Internal Medicine), PUBLIC HEALTH, patient activation

## Abstract

**Abstract:**

**Objectives:**

Patient activation (PAct)—a measure assessing an individual’s perceived knowledge, skills and confidence in managing their health and well-being—is often used to personalise and evaluate care, although its causal link to self-management behaviours (SMBs) and clinical outcomes remains uncertain. We aimed to synthesise the evidence on the causal association between PAct, SMBs and clinical outcomes in type 2 diabetes (T2D).

**Design:**

Systematic review and narrative synthesis of data summarised in a harvest plot.

**Data sources:**

We searched Medline, Embase, CENTRAL, PsycInfo, Web of Science and CINAHL up to April 2024 for relevant English articles.

**Eligibility criteria:**

We included studies of any quantitative design that reported on the association of PAct with clinical outcomes or SMBs in adult patients with T2D.

**Data extraction and synthesis:**

Two independent reviewers were involved, and any disagreements were discussed and resolved collaboratively. Risk-of-bias (RoB) was assessed using an adapted RoB Assessment Tool for Nonrandomised Studies. Levels of evidence were evaluated for each T2D-related outcome.

**Results:**

We identified 21 studies published between 2009 and 2023, including 15 cross-sectional studies and no randomised controlled trials. Eleven studies were conducted in the USA. Seventeen studies used the Patient Activation Measure questionnaire. There is moderate evidence that higher PAct scores are associated with better glycated haemoglobin levels (studies reporting on this association, n=14). There is very limited evidence that PAct improves diet (n=5) and physical activity (n=6). All other clinical outcomes and SMBs had inconclusive results due to either inconsistent or insufficient evidence, or both.

**Conclusion:**

A causal relationship between PAct, clinical outcomes and SMBs in T2D cannot be established due to inconsistent evidence and a lack of high-quality studies. Thus, the use of PAct scores as a tailoring tool and an outcome measure in healthcare services requires further evaluation.

**PROSPERO registration number:**

CRD42021230727.

**STRENGTHS AND LIMITATIONS OF THIS STUDY**

- By using a sensitive search strategy and including all study designs, this review comprehensively analyses the evidence for patient activation in type 2 diabetes (T2D).
- We derive levels of evidence, which incorporate the strength of the study design/analysis, the study quality, sample size and consistency of the findings for a broad range of clinical outcomes and self-management behaviours, which provide a thorough assessment of causal assumptions for each T2D-related outcome.
- The scarcity of studies for certain outcomes limited our ability to synthesise evidence and evaluate causal assumptions.
- The high heterogeneity across studies made it inappropriate to conduct a meta-analysis and therefore the magnitude of associations could not be quantified.

## Introduction

 A recent study highlighted that the National Health Service (NHS) in the UK spends about £14 billion per year on diabetes, the majority (approximately 60%) on complications.^1^ This is projected to increase to over £23 billion in 2036.^1^ Therefore, strategies to curb the largely preventable complications of type 2 diabetes (T2D) are essential to reduce long-term costs and improve patient outcomes.

Effective management through lifestyle modifications, medication adherence and regular reviews can significantly mitigate T2D complications.^2^ A key component is the optimisation of self-management behaviours (SMBs), which is associated with sustained control of risk factors for complications such as blood glucose, blood pressure and cholesterol.

Patient activation (PAct) refers to an individual’s knowledge, skills and confidence in managing their health and well-being, and is theorised to be a fundamental component in supporting effective SMBs.^3^ PAct is measured using various tools, like the Patient Activation Measure (PAM),^4^ which is the most prevalent method and also used within the UK NHS. Other general instruments like the Patient Assessment of Chronic Illness Care (PACIC)^5^ and T2D-specific tools like the Influence and Motivation for Patient ACTivation in Diabetes care^6^ are also employed in certain settings.

PAct strategies have been incorporated in the NHS’ Comprehensive Model for Personalised Care, developed to tackle the increasing demographic and financial strains placed on the NHS.^3 7^ For instance, supported self-management involves measuring a patient’s PAct and tailoring approaches based on the results as well as offering interventions to increase PAct.^7^ PAct is also used as a performance measure in the NHS.^8^ The underlying assumptions are that PAct levels are predictive of health outcomes and increases in PAct lead to improvements in health outcomes.^9^ Therefore, it is important to investigate the evidence for a causal link between PAct and T2D-related outcomes to justify its widespread use as a tailoring tool and an outcome measure.

Empirical evidence suggests that people with higher PAct are more inclined to engage in preventive actions, including attending regular check-ups,^9 10^ adopting healthier lifestyles, such as maintaining a balanced diet and engaging in consistent physical activity and avoiding harmful behaviours such as smoking.^411^,^16^ Studies also suggest that people with higher PAct are more likely to have better clinical outcomes, including body mass index (BMI), glycated haemoglobin (HbA_1c_), blood pressure and cholesterol levels. However, the findings are inconsistent and largely derived from cross-sectional studies, raising the possibility of reverse causality.^1217^,^19^

Among people living with T2D, evidence for an association between PAct and SMBs is highly variable. Some studies report associations with physical activity^20^ and medication adherence,^13 21^ while others found no association with smoking status^20 22^ and attendance at routine appointments.^20^

PAct has been shown to predict poorer health outcomes in people with diabetes two years later,^17^ potentially enabling healthcare providers to identify high-risk individuals early and target proactive management strategies. However, the evidence base is inconclusive. Some studies report favourable associations between PAct and HbA_1c_,^17 18 21 23^ blood pressure,^12 21 23^ low-density lipoprotein (LDL)^12 21 23^ and high-density lipoprotein (HDL),^23^ while other studies report no association with HbA_1c_,^12 24^ blood glucose levels,^22^ blood pressure^22^ and LDL.^17^ Overall, the evidence base on PAct, SMBs and clinical outcomes in T2D is inconsistent. Evidence from longitudinal studies for a causal link between PAct and diabetes-relevant outcomes is limited. In one study of mixed conditions (including T2D), Greene *et al* found that higher PAct was predictive of 9 out of 13 better health outcomes and lower costs two years later.^25^

Systematic reviews of PAct interventions in T2D present moderate evidence for small improvements in HbA_1c_ and SMBs.^26 27^ There is limited evidence for effects of PAct interventions on other clinical outcomes such as blood pressure, LDL and body weight.^27^ However, the included interventions are complex and often involve multiple components, making it difficult to ascertain whether effects are due to changes in PAct or other factors. A random-effects meta-analysis found no significant changes in PAct scores, HbA_1c_ or BMI between intervention and control groups.^28^ Overall, these reviews, while providing some evidence of the effectiveness of interventions targeting PAct, do not provide insights on whether measures of PAct (eg, the PAM)^11^ are predictive of, and causally linked with, T2D outcomes. Given the widespread use of PAct measures in healthcare services, it is important to explore the validity of PAct as a predictive measure of outcomes.

We aimed to critically appraise and synthesise evidence on the association between PAct and SMBs and clinical outcomes in adults with T2D and address the following questions: (1) What is the evidence of the association between PAct and clinical outcomes of adults with T2D?; (2) What is the evidence of the association between PAct and SMBs of adults with T2D?; (3) What is the level of evidence available for the associations observed and is this sufficient to suggest a causal role of PAct in improving clinical outcomes and SMBs?

## Methods

The protocol for this review has been published.^29^ Initially, the population included diabetes and related metabolic disorders. Since a scoping review identified sufficient studies, we decided to focus on T2D. Deviations from the protocol are summarised in online supplemental table S1.

### Data sources and search strategy

We searched Medline, Embase, CENTRAL, PsycInfo, Web of Science and CINAHL using Medical Subject Headings and keywords related to the terms *patient activation* and T2D. The search strategies are included in online supplemental tables S2–S7. We included broad terms related to diabetes in the initial search strategy to ensure a comprehensive retrieval of all relevant literature, given the potential overlap in research across different types of diabetes. Subsequently, we excluded studies that did not specifically focus on T2D.

We initially included PACIC^30^ studies because the tool purports to measure PAct. However, on review, we decided to exclude studies reporting PACIC scores because the PACIC questionnaire focuses on how care experiences support patients’ SMBs,^31^ instead of directly measuring PAct.

All the databases were searched up to 25 April 2024. We included grey literature only if a full-text article was available.

### Selection criteria

Studies were eligible if they utilised any PAct measure and investigated the relationship between PAct and T2D-related outcomes, or if they evaluated the impact on these outcomes of interventions specifically designed to enhance PAct. Detailed eligibility criteria are provided in table 1.

**Table 1:** Inclusion and exclusion criteria for study selection

Screening parameter	Inclusion criteria	Exclusion criteria
Population	Adults (≥18 years old) with T2D	Any other disease (eg, pre-diabetes, type 1 diabetes and gestational diabetes) Age<18 years old
Exposure	Studies that reported a measure of PAct (eg, Patient Activation Measure (PAM) or other PAct measures)	Studies that evaluated related constructs such as confidence or self-efficacy
Outcomes	Both self-reported and objectively measured outcomes were included Clinical outcomes HbA_1c_ level/ glycaemic control Systolic blood pressure/diastolic blood pressure Low-density lipoprotein (LDL)/ high-density lipoprotein (HDL)/ total cholesterol Serum triglycerides Body mass index (BMI)/body weight Self-management behaviours Overall self-management score Outcomes related to diet (e.g. fruit/ vegetable consumption, following a low-fat diet) Outcomes related to physical activity (e.g. step counts, following a regular exercise schedule, frequency of physical activity) Smoking status Outcomes related to alcohol consumption (e.g. alcohol consumption, frequency or amounts)	All other outcomes not listed
Study design	Original primary research articles All study designs, including cross-sectional, longitudinal and intervention (e.g. RCTs, pre–post comparison studies) were included if they reported the association between PAct and T2D-related outcomes. Note: For this review, we classified study design based on how it reports the relationship between PAct and T2D-related outcomes. For instance, if an RCT did not report T2D-related outcomes for each intervention group separately and reported pooled temporal associations between PAct and T2D-related outcomes instead, it was treated as a cohort study.We included intervention studies that report intervention effects on PAct and effects on other specified outcomes but do not directly report on the association of PAct and outcomes if the interventions fulfilled the following criteria: the intervention explicitly targets PAct or is described as enhancing patients’ knowledge, confidence and skills for self-management (as opposed to interventions targeting different related constructs such as self-efficacy); and Increasing PAct is a pivotal, main component of the intervention; and PAct was measured; and the intervention increased PAct scores (PAct measured post-intervention is significantly higher compared with the control group)	Study protocols Editorials Literature reviews/meta-analyses Qualitative studies Studies not reporting on empirical data Interventions that did not significantly increase PAct scores (this indicates a shortcoming in its premise or implementation, making it irrelevant to the analysis because it does not contribute to our understanding of how PAct influences T2D-related outcomes). Interventions where PAct components form part of a complex intervention with other components
Comparators	For intervention studies, any type of comparator was eligible. This included observational studies or intervention studies with no comparator, e.g. pre–post studies.	–
Language and date	Only articles in English were included There were no restrictions on publication dates	Articles not in English

HbA_1c_, glycated haemoglobin; PAct, patient activation; RCTs, randomised controlled trials; T2D, type 2 diabetes.

### Data management and selection process

The citations retrieved from the databases were deduplicated and imported into Covidence systematic review software. A second reviewer independently screened 10% of the citations at each stage. Any disagreements were discussed and resolved collaboratively. We assessed interrater reliability using Cohen’s Kappa^32^ and percentage agreement^33^ via Covidence.

### Data extraction

Data on study design, population, sample size, intervention details (if applicable), outcome assessment methods and the reported association between PAct and T2D-related outcomes were extracted using a data extraction sheet (refer to the online supplemental appendix) and were independently verified by a second reviewer. Missing or unclear data on associations were omitted from the analysis.

### Risk-of-bias/quality appraisal

To assess the risk-of-bias (RoB), we intended to use the revised Cochrane RoB 2 tool for randomised controlled trials (RCTs),^34^ but no RCTs were identified. For other study designs, we supplemented the RoB Assessment Tool for Nonrandomised Studies^35^ with items from the Quality Assessment Tool for Observational Cohort and Cross-sectional studies from the National Heart, Lung and Blood Institute.^36^

The ‘measurement of exposure’ component, which scores objectively measured exposures higher than self-reported measures, was omitted from the RoB assessment to streamline the evidence evaluation. Since the exposure across all studies was PAct, which is measured exclusively through questionnaires, including this criterion would not have contributed to differentiating between high and low-quality studies. We did, however, consider the limitations of self-reported measures in our interpretation of the results.

Two independent reviewers appraised each study and discussed any discrepancies until they reached a consensus. We assigned each study an overall quality rating of high, low or some concerns based on RoB assessments, which was then used to determine the level of evidence for each T2D-related outcome.

### Data synthesis and analysis

We used a Preferred Reporting Items for Systematic Reviews and Meta-analyses (PRISMA) diagram to describe the study selection process.^37 38^ It was inappropriate to conduct a meta-analysis due to heterogeneity in methods. Therefore, we conducted a narrative synthesis by summarising the information in a harvest plot produced using Microsoft PowerPoint 2021 (V.16, Microsoft Corporation).

We adapted the harvest plot from Ogilvie *et al*’s^39^ approach. Each study is represented by a bar and the associations were categorised into negative/none/positive based on statistical significance (p<0.05). The bar heights denote the sample size, colours represent the quality and patterns indicate the study design (see online supplemental table S9). A large study was defined as one with >250 participants or a sample size justified by a power calculation, while studies with ≤250 participants were categorised as small.^40^ For the harvest plot, ‘large’ was further differentiated into ‘large’ (>250) and ‘very large’ (≥1000) to provide further detail.

For each T2D-related outcome, we specified a null hypothesis (that there is no association between PAct and the outcome) and an alternative hypothesis (that higher PAct is associated with a better outcome). If higher PAct was associated with a worse T2D-related outcome, this was classified as against the hypothesised direction of association. The direction of association that corresponds to better T2D-related outcomes is defined in online supplemental table S8.

A key output of this review is an evaluation of the evidence available for inferring a causal association between PAct and T2D-related outcomes. For each outcome, we determined the ‘level of evidence’ for a causal association with PAct, using the flowcharts depicted in online supplemental figures S1 and S2. The ‘level of evidence’ is a composite assessment synthesised from the strength of the study design/analysis, the study quality, sample size and consistency of the findings, adapted from an approach used in a prior systematic review^40^ to include the strength of the study design/analysis and the sample size.

## Results

### Search results

**Figure 1:**
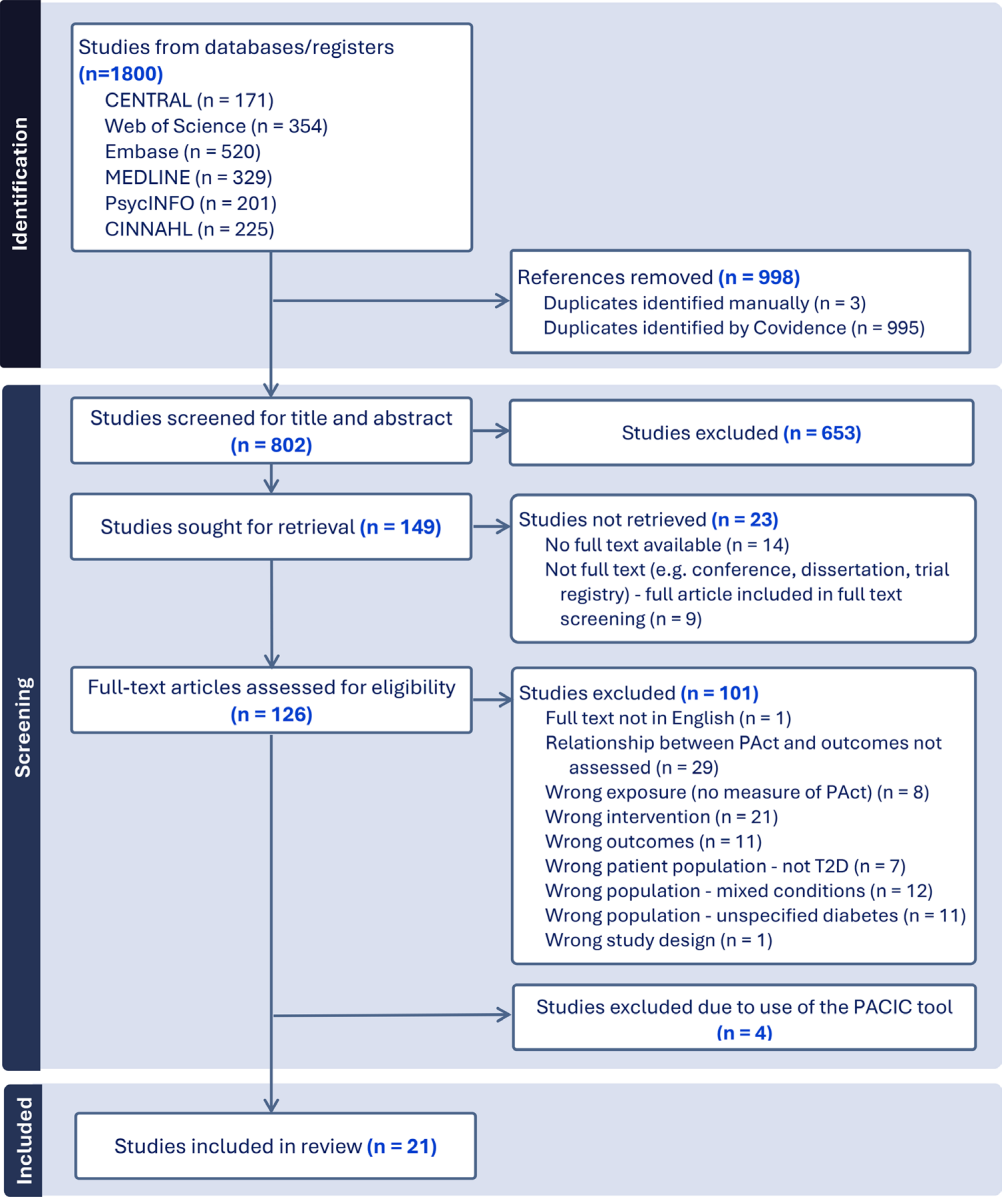
The PRISMA flow diagram for the systematic review detailing the database searches, number of abstracts screened, full-text articles retrieved and the reasons for exclusion. This diagram was modified from the Covidence output. PACIC, Patient Assessment of Chronic Illness Care; PRISMA, Preferred Reporting Items for Systematic Reviews and Meta-analyses.

The PRISMA flowchart in figure 1 shows the search and study selection.^41^ We excluded four papers because they used the PACIC tool.^42^,^45^ We extracted information from 21 studies for the analysis.

### Methodological quality

The inter-rater reliability as measured by Cohen’s kappa was 0.51 for the title and abstract screening (with 79.3% agreement), and 0.66 for full-text screening (with 86.7% agreement). However, during conflict resolution, it was observed that reviewers had similar opinions on 12 out of 17 articles at the title and abstract stage and 6 out of 8 articles during the full-text review (most discrepancies pertaining to reasons for exclusion, where several reasons applied). Full consensus was reached after discussion.

### Study characteristics

The studies collectively included a total of 13 416 participants, with a mean age varying between 49.4 and 73.6 years (table 2). Analyses were cross-sectional in 71% of studies and PAM-13 was the most frequently used tool for assessing PAct. Eleven studies were conducted in the USA. HbA_1c_ was the most frequently measured clinical outcome, while physical activity and smoking were the most reported SMBs. Outcomes like LDL, body weight and alcohol consumption were infrequently reported. The follow-up period for longitudinal and intervention studies ranged from 3 months to 2 years.

**Table 2:** Summary of studies investigating the association between PAct and T2D-related outcomes

Study ID	Publication author (year)	Study design for analysis (country)	Participant details; number (age, sex)	Setting	Patient activation measure	T2D-related outcomes
1	Almutairi *et al* (2023)^48^	Pre–post intervention study (Saudi Arabia)	82 (mean age 51.3 years, 39% male)	Primary care	PAM-13	HbA_1c_, blood pressure, cholesterol, triglycerides, BMI, diet, physical activity, medication adherence
2	Almutairi *et al* (2023)^62^	Cross-sectional (Saudi Arabia)	398 (mean age 53.2 years, 54.9% male)	Primary care	PAM-13	HbA_1c_, blood pressure, cholesterol, triglyceride, BMI, diet, physical activity, medication adherence, smoking
3	Arvanitis *et al* (2020)^6^	Cross-sectional (USA)	300 (mean age 63.2 years; 43.7% male)	Internal medicine clinics	IMPACT-D	HbA_1c_, systolic blood pressure
4	Aung *et al* (2015)^63^	Cohort (Australia)	3040 (mean age 64.6 years; 55% male)	Community	PAM-13	BMI
5	Glenn *et al* (2019)^64^	Cross-sectional (USA)	58 (mean age 59 years, 27% male)	Community	PAM-10	HbA_1c_
6	Hendriks *et al* (2016)^65^	Cross-sectional (Netherlands)	1615 (mean age 68 years, 54.1% male)	Primary care	PAM-13	HbA_1c_, BMI, smoking
7	Kato *et al* (2020)^66^	Cross-sectional (Japan)	209 (mean age 60.2 years, 80% male)	Hospital outpatient units	PAM-13	HbA_1c_, BMI
8	Kim *et al* (2021)^67^	Cross-sectional (South Korea)	155 (mean age 51.5 years, 47.4% male)	Hospital ambulatory care unit	PAM-13	Self-care activities
9	Ledford *et al* (2012)^68^	Cross-sectional (USA)	130 (mean age 59.8 years, 52.3% male)	Family medicine clinic	PAM-13	HbA_1c_, BMI, physical activity
10	Mayberry *et al* (2010)^69^	Cross-sectional (USA)	48 (66.7%<65 years, 57% male)	Primary care	PAM-13	HbA_1c_, overall self-management score
11	Michaud *et al* (2018)^70^	Pre–post intervention (USA)	955 (mean age 60 years, 45% male)	Hospital	PAM-13	HbA_1c_
12	Parchman *et al* (2010)^21^	Cross-sectional (USA)	141 (mean age 57.7 years, 39% male)	Primary care	Lorig communication scale	Medication adherence
13	Rask *et al* (2009)^20^	Cross-sectional (USA)	287 (mean age 51.5 years, 41.1% male)	Diabetes clinic	PAM-13	Smoking, exercise, diet
14	Regeer *et al* (2022)^49^	Longitudinal (Netherlands)	603 (mean age 62.8 years, 46.6% male)	Primary care	PAM-13	BMI, weight, HbA_1c_, exercise behaviour, general diet
15	Rogvi *et al* (2012)^18^	Cross-sectional (Denmark)	1081 (mean age 64.3 years, 65% male)	Specialist diabetes clinic	PAM-13	HbA_1c_
16	Shah *et al* (2015)^50^	Longitudinal (USA)	60 (mean age 49.4 years, 32% males)	Zuni Indian Community	PAM-13	BMI, HbA_1c_, cholesterol, triglycerides
17	Stuart *et al* (2021)^71^	Cross-sectional (USA)	940 (83% aged≥65 years, 41% male)	Medicare beneficiaries	Williams/Heller Segmentation Screening Tool (SST)	Medication adherence
18	Su *et al* (2019)^51^	Longitudinal (USA)	1354 (mean age 59.6 years, 45.1% male)	Hospital	PAM-13	HbA_1c_
19	Van Vugt *et al* (2018)^72^	Cross-sectional (Netherlands)	1189 (mean age 66 years, 58.8% male)	General practices and outpatient clinics	PAM-13	HbA_1c_, BMI, LDL, blood pressure, smoking, alcohol
20	Zhang *et al* (2023)^73^	Cross-sectional (China)	200 (mean age 73.6 years, 49% male)	Community hospital	PAM-13	Diabetes self-management ability, diet, exercise, smoking status
21	Zheng (2018)^74^	Cross-sectional (USA)	571 (mean age 72.4 years, 43% male)	Medicare beneficiaries	PAct Supplement in the Medicare Current Beneficiary Survey	BMI, smoking, medication adherence

BMI, body mass index; HbA_1c_, glycated haemoglobin; HDL, high-density lipoprotein; IMPACT-D, Influence and Motivation for Patient ACTivation in Diabetes care; LDL, low-density lipoprotein; PAct, patient activation; PAM-10/13, Patient Activation Measure; T2D, type 2 diabetes.

### Quality appraisal

The RoB results are summarised in online supplemental figure S3. Overall, 7 (33%) studies were low quality, 4 (19%) had some concerns and 10 (48%) were high quality. For the overall assessment, confounding and selection bias were the primary criteria for quality assessment, with the other factors considered collectively to arrive at the final judgement. Most studies were low quality due to selection bias and failing to adjust for potential confounders. Particularly in the context of this review, factors such as age, disease duration and comorbidities can influence both PAct and outcomes, so should be accounted for in the analyses. Furthermore, the reliance on self-reported questionnaires to measure PAct predisposes the studies to selection bias, which is exacerbated by convenience sampling or the selective inclusion of patients with digital tools, potentially limiting generalisability.

### Levels of evidence for an association between PAct and clinical outcomes and SMBs

**Figure 2:**
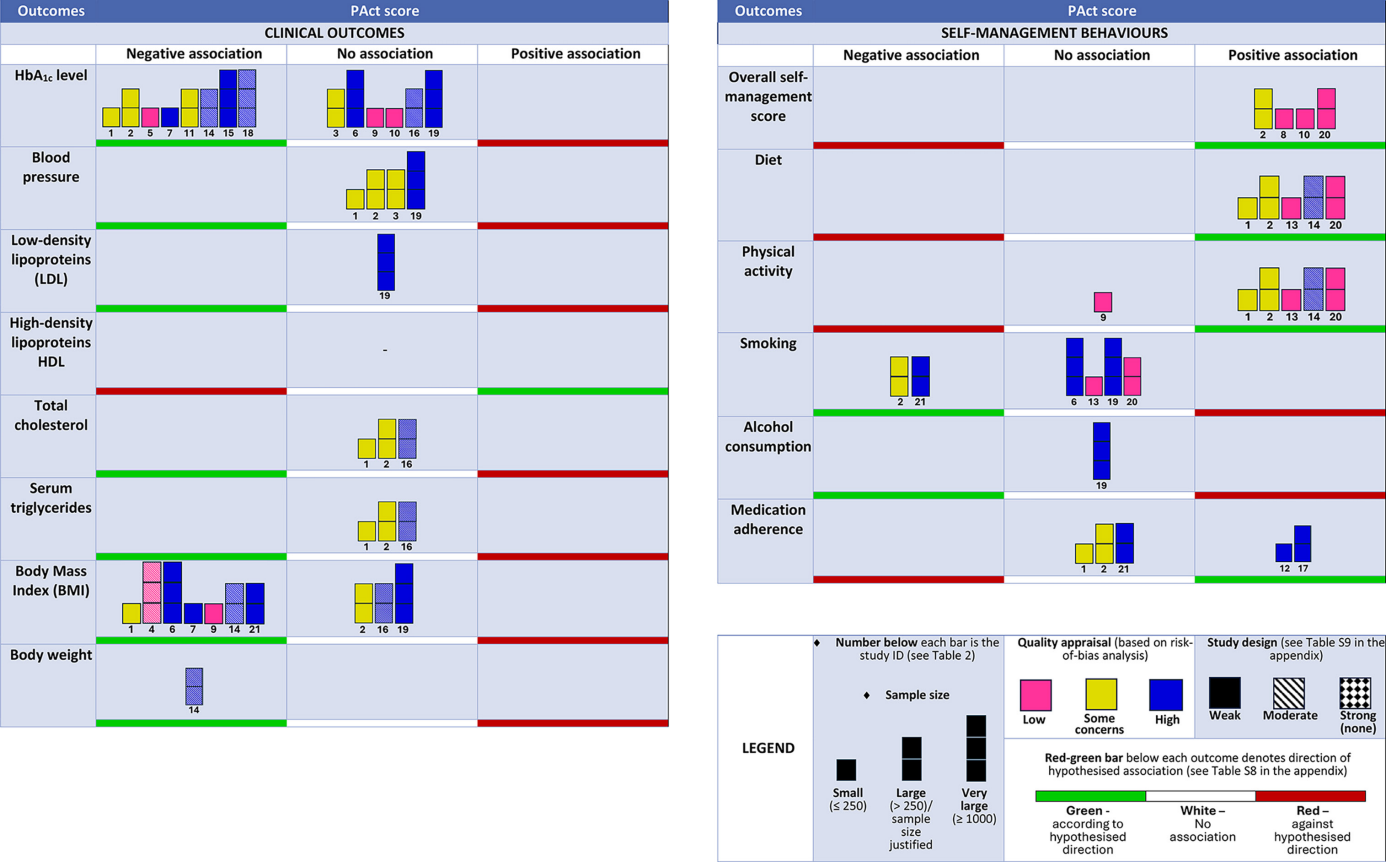
Harvest plot of evidence for association between PAct scores and T2D-related outcomes. Each study is represented by a bar. HbA_1c_, glycated haemoglobin; PAct, patient activation; T2D, type 2 diabetes.

The findings of the included studies are illustrated in the harvest plot in figure 2. The harvest plot highlights that while several studies examine outcomes like HbA_1c_, BMI, physical activity and smoking, few studies reported LDL, HDL, alcohol consumption and overall self-management scores. Importantly, all studies reported results in line with the hypothesised direction or indicated no association. None of the findings of studies contradicted the expected direction of association, that is, worse T2D-related outcomes with higher PAct.

### Summary of evidence for an association between PAct scores and each outcome

Tables3  4 summarise the levels of evidence for a causal relationship between PAct and each T2D-related clinical outcome and SMB, respectively. Due to the lack of large, high-quality RCTs, no outcome attained a ‘strong’ or ‘very strong’ level of evidence for causality.

**Table 3:** Levels of evidence for a causal effect of patient activation (PAct) on T2D-related clinical outcomes

Outcome	Evidence for PAct scores	Description
HbA_1c_/glycaemic control	Moderate evidence of favourable association	14 studies reported on the association between HbA_1c_ and PAct scores.^618 48^,^51 62 64^ This included two large high-quality studies with moderate design,^49 51^ one large high-quality cross-sectional study^18^ and one small high-quality cross-sectional study that reported lower HbA_1c_ levels with higher PAct scores.^66^ One large high-quality study with moderate design^50^ and two large high-quality cross-sectional studies^65 72^ reported no association between HbA_1c_ and PAct scores. Moderate evidence that higher PAct scores are associated with better HbA_1c_ values because two out of three high-quality studies with moderate designs and large sample sizes reported this finding.^49 51^
Blood pressure	Inconclusive	All four studies reported no association between blood pressure and PAct scores^6 48 62 72^ and had uniformly weak designs Only one was high-quality.^72^ Inconclusive evidence
Low-density lipoprotein (LDL)	Inconclusive	Only one study high-quality study with a weak design assessed the association between LDL and PAct scores and reported no association.^72^ Inconclusive evidence
High-density lipoprotein (HDL)	No evidence	No studies reported on the association between HDL and PAct scores.
Total cholesterol and serum triglycerides	Very limited evidence of no association	Three studies evaluated the association between total cholesterol and serum triglycerides with PAct scores,^48 50 62^ all reported no association. The only high-quality study had a moderate design and large sample size,^50^ while the remaining studies were low-quality and had weak designs, including one with a large sample size.^62^ Very limited evidence that PAct has no association with total cholesterol and serum triglycerides.
Body mass index (BMI)	Inconclusive	Four high-quality studies reported lower BMI with increased PAct scores,^49 65 66 74^ three of which had large sample sizes,^49 65 74^ including one with a moderate design^49^ and the rest with weak designs.^65 66 74^ One large low-quality study with moderate design reported lower BMI with high PAct scores.^63^ Two other high-quality studies, one large with a weak design,^72^ and one large study with a moderate design^50^ found no association between PAct scores and BMI. Inconclusive evidence because both the large high-quality studies with moderate designs^49 50^ are not in agreement.
Body weight	Inconclusive	Only one large high-quality study with moderate design reported a lower body weight with higher PAct scores.^49^ Inconclusive evidence

HbA_1c_, glycated haemoglobin; PAct, Patient activation; T2D, type 2 diabetes.

**Table 4:** Levels of evidence for a causal effect of patient activation (PAct) on T2D-related self-management behaviours

Outcome	Evidence for PAct scores	Description
Overall self-management scores	Inconclusive	All the studies reporting overall self-management scores were low-quality^62 67 69 73^ Although with weak design, all four consistently reported higher overall self-management scores with higher PAct scores Inconclusive evidence
Diet	Very limited evidence of favourable association	All the studies available reported a more favourable diet with higher PAct scores,^20 48 49 62 73^ which indicate consistent results These studies measured both general diet (healthy eating)^20 48 49 62 73^ and specific diet (fruits, vegetables and high-fat foods).^62 73^ Only one study was large and high-quality with a moderate design^49^ All the others were low-quality. Very limited evidence of favourable association between diet and higher PAct scores
Physical activity	Very limited evidence of favourable association	All studies that investigated diet also reported higher physical activity with higher PAct scores^2062 65 72^,^74^ with the same spread of results One addition is a small, low-quality study with a weak design that showed no association.^68^ Very limited evidence of favourable association between PAct scores and physical activity
Smoking	Very limited evidence of no association	All the studies that investigated the association between smoking and PAct scores had weak designs^2065 72^,^74^ Two large high-quality studies reported no association between smoking and PAct scores.^65 72^ Two large studies, one high-quality^74^ and one low-quality^62^ showed lower levels of smoking with increased PAct scores. Very limited evidence of no association between smoking and PAct scores
Alcohol consumption	Inconclusive	Only one large high-quality study with a weak design^72^ reported no association between alcohol consumption and PAct scores. Inconclusive evidence
Medication adherence	Inconclusive	All the studies that investigated medication adherence had weak designs.^21 48 62 71 74^ The methods used to measure medication adherence were proportion of days covered,^71 74^ Morisky scale^21^ and the Summary of Diabetes Self-Care Activities (SDSCA) tool.^48 62^ Among the high-quality studies, two (one large and one small) reported higher medication adherence with higher PAct,^21 71^ whereas one large study reported no association.^74^ Inconclusive evidence

PAct, Patient activation; T2D, type 2 diabetes.

There is moderate evidence that high PAct results in better HbA_1c_ values, and very limited evidence that there is no association between PAct and total cholesterol as well as serum triglycerides. Evidence for all other clinical outcomes is inconclusive. For SMBs, the highest level of evidence is ‘very limited’ for a positive effect of PAct scores on diet and physical activity. There is similarly very limited evidence that PAct scores have no association with smoking status. Evidence for all other SMBs is inconclusive.

## Discussion

### Summary of findings

To our knowledge, this is the first comprehensive systematic review of evidence concerning PAct and T2D-related outcomes that encompasses a diverse array of study types and a broad range of clinical outcomes and SMBs. Although there were several studies reporting on the associations between PAct and T2D-related outcomes, the evidence for a causal association is limited. There was insufficient evidence to establish causality for any of the T2D-related outcomes. Moreover, even for frequently reported outcomes like BMI, the findings were inconsistent, leaving uncertainty about whether increasing PAct improves BMI or has no effect. Overall, the findings indicate that a causal relationship between PAct and T2D-related outcomes cannot be inferred. The insufficient and inconsistent results highlight a significant gap in our understanding of the effect of PAct on T2D-related outcomes. There was insufficient evidence to establish causality overall primarily due to the predominance of cross-sectional studies in the review, with no RCT meeting the inclusion criteria. Cross-sectional studies offer weak causal inference as they only provide correlational evidence without addressing temporal relationships or controlling for unmeasured confounders. Although RCTs are a stronger study design for inferring causality due to their ability to establish temporality and better control unmeasured confounders, the lack of RCTs necessitates reliance on weaker evidence. Moreover, the inconsistent findings for T2D-related outcomes such as BMI and medication adherence, along with scarce evidence for other T2D-related outcomes like blood pressure, LDL, HDL and total cholesterol constrained the synthesis of evidence on causal associations between PAct and T2D-related outcomes.

Two other RCTs were excluded because there was no difference in PAct scores between the intervention and control groups,^46^ or the control group had higher PAct scores than the intervention group.^47^ Both these studies exemplify significant limitations in the evidence, demonstrating that some PAct interventions do not appear to increase PAct scores, and that clinical outcomes can improve independent of changes in PAct scores. There were two pre–post intervention studies included in this review. One showed no associations for blood pressure, LDL, total cholesterol and medication adherence^48^ with PAct. This could be attributable to variability in the time required for PAct interventions to translate to SMBs and subsequently, clinical outcomes. The optimal duration for an intervention to achieve sustained increases in PAct and thereby SMBs remains uncertain and current interventions vary widely in their duration, approach and measures. Thus, the study could have reported no associations because the duration of the intervention—3 months—was insufficient to produce a meaningful change in T2D-related outcomes, but this requires further investigation.

This review found moderate evidence that higher PAct is associated with lower HbA_1c_, derived primarily from three cohort studies.^49^,^51^ Two report lower HbA_1c_ with higher PAct,^49 51^ while one study found no significant association.^50^ Therefore, although there is some evidence that changes in PAct lead to better HbA_1c_ values, the evidence for causality is scarce and mixed. Nevertheless, other systematic reviews also report a similar association between PAct and HbA_1c_.^26 27 52^ For instance, Bolen *et al*’s meta-analysis^27^ also found moderate evidence for small improvements in HbA_1c_ with higher PAct. Furthermore, they report low evidence for small improvements in blood pressure and body weight, and very low evidence for small improvements in LDL and triglyceride levels. Bolen *et al* also found low evidence that PAct scores are not associated with HDL. In comparison, this review found inconclusive results for blood pressure, LDL and body weight, and very limited evidence for no association with serum triglycerides, where most of the studies showed no associations with PAct. This could be because Bolen *et al* assessed the effect of PAct interventions (broadly defined with significant overlap with other behavioural interventions), whereas we specifically investigated causal associations between PAct and T2D-related outcomes.

Interestingly, our review identified some, though very limited, evidence of better diet and physical activity levels with higher PAct scores. This relates to Samdal *et al*’s meta-regression, which found that to support physical activity and diet, counselling techniques should prioritise self-monitoring, goal-setting and patient-autonomy,^53^ which essentially refer to PAct. More evidence is needed to determine whether the weak positive associations observed are due to limitations within the existing research, or if they indicate a broader challenge of achieving significant improvements in diet and physical activity with higher PAct. In a mixed condition meta-analysis of RCTs investigating the effect of PAct-interventions, Lin *et al* found significant improvements in HbA_1c_, body weight and LDL.^52^ Although their findings for HbA_1c_ align with ours, we found inconclusive evidence for body weight and LDL. These differences could be attributed to Lin *et al*’s inclusion of people with mixed conditions (eg, LDL might be more of a concern for people with cardiovascular disease compared with T2D). Differences may also be attributable to the differing aims of the reviews: Lin *et al*’s review included complex interventions that targeted PAct alongside other psychosocial/ behavioural constructs, hence specific effects of PAct cannot be isolated. In contrast, this review sought to specifically assess the effects of PAct on outcomes. Our findings of inconclusive evidence for most T2D-related outcomes align with Kearns *et al*’s systematic review of PAM-tailored interventions, which found that the scarce studies reporting clinical outcomes and SMBs and the inconsistency of findings limit the generalisability of the benefits of these interventions.^54^ Notably, while many studies concur that increased PAct scores correlate with improved HbA_1c_ levels, there is no strong evidence for causation, which necessitates more nuanced research to disentangle the specific components of PAct interventions that drive the observed improvements and to establish a causal relationship between PAct scores and T2D-related outcomes.

### Implications for research and practice

This review found moderate evidence for a causal association between PAct and HbA_1c_, with most other clinical outcomes remaining inconclusive. In terms of SMBs, there is very limited evidence that PAct leads to better diet and increased physical activity. Taken together, this suggests that relying on PAct interventions to improve T2D care might be premature, as the causal associations and relationships between PAct and T2D-related outcomes are not fully understood. The tepid evidence for causal associations between PAct and T2D-related outcomes needs to be considered by policymakers. This review suggests that most of the evidence for assuming a causal relationship is very limited or inconclusive. Therefore, use of PAct in healthcare services for T2D patients may not currently be justified since it has, at best, moderate evidence of a favourable association with one clinical outcome, and most other T2D-related outcomes are inconclusive. The use of PAct as a tailoring tool and an outcome measure becomes questionable if it does not lead to meaningful measurable benefits.

The inconsistent evidence on T2D-related outcomes observed could also be due to other unmeasured factors that mediate or moderate the relationship between PAct and outcomes, such as health literacy, use of technology, socioeconomic status, mental health and psychosocial support, which vary across studies and may lead to misconstruing correlations as causal associations. Conversely, PAct could be a crucial component to increasing SMBs that greatly improve patient outcomes, but something in the way we measure PAct or design and evaluate interventions might be obscuring these associations. Crucially, none of the studies report that increased PAct scores are associated with worse T2D-related outcomes. This assures us that while we do not know definitively if increasing PAct will improve T2D-related outcomes, it is unlikely to harm patients by decreasing them. However, long-term effects of PAct interventions cannot be assessed due to the lack of long-term cohort and intervention studies.

Without precise and consistent estimates of benefits, policymakers cannot confidently determine whether the resources and efforts required to implement PAct strategies are justified. For example, although this review indicates moderate evidence for better HbA_1c_ scores, the magnitude of improvement is unclear.

### Future work

Future RCTs, and mediation and pathway analyses might elucidate causal relationships between PAct and T2D-related outcomes. For instance, diet and physical activity influence both weight loss^55^ and HbA_1c_.^56^ Weight loss also has a dose-dependent effect on HbA_1c_ levels.^57^ Therefore, it is essential to determine whether the overall effect of PAct on HbA_1c_ observed is mediated through diet and physical activity, if weight loss plays a role, or if alternative pathways are involved. This could steer future PAct interventions to elements that it can improve, besides suggesting its applicability in specific patient subgroups. Moreover, long-term follow-up beyond 24 months is required to assess the sustained impact of PAct interventions.

### Strengths and limitations

This study synthesises the evidence for a causal relationship between PAct and several T2D-related outcomes, offering several novel insights. First, by including all study types, it provides a comprehensive overview of the existing evidence, which reveals that most assumptions on PAct’s effectiveness rely on correlational evidence from cross-sectional analyses. Furthermore, unlike other narrative syntheses, this review systematically assesses the evidence for each T2D-related outcome, highlighting gaps and inconsistencies in the evidence base.

Some limitations of this study are inherent to systematic reviews, notably publication bias.^58^ This can lead to conclusions based on a biased subset of the totality of evidence, which could possibly inflate the association between PAct scores and T2D-related outcomes. A limitation of the evidence included in this review is that all PAct scores and some SMBs such as diet, physical activity and smoking status are obtained from questionnaires, which may be subject to error, non-response bias, selection bias, recall bias and social desirability bias.^59^,^61^ Significant factors associated with T2D-related outcomes—such as patient demographics, health literacy, healthcare access and utilisation, healthcare providers’ engagement and skill, patient support systems, behavioural and psychological factors—may also be confounded with other variables in the studies, which could potentially obscure the associations observed. Despite these limitations, this review offers a critical evaluation of the evidence linking PAct scores to outcomes and highlights significant gaps in the current literature.

## Conclusion

Currently, a causal relationship between PAct and T2D-related outcomes cannot be established. There is moderate evidence that higher PAct is associated with better HbA_1c_ values. Most other outcomes remain inconclusive. A key observation is that most studies are cross-sectional, and some PAct interventions do not significantly increase PAct compared with the control group. This highlights the critical need for future research to generate more robust, high-quality RCTs and mediation analyses to establish clearer causal relationships so that more informed decisions on resource allocations can be made. In summary, this review calls for a re-evaluation of current intervention strategies and a concerted effort to develop a more robust evidence base for employing PAct strategies in healthcare systems.

## Supplementary material

10.1136/bmjopen-2024-095456 online supplemental file 1

10.1136/bmjopen-2024-095456 online supplemental file 2

## Data Availability

All data relevant to the study are included in the article or uploaded as supplementary information.
