# Supplementary material for: Association between patient activation, self-management behaviours and clinical outcomes in adults with type 2 diabetes: a systematic review with narrative synthesis
Source: BMJ Open. 2025 May 27;15(5):e095456. doi: 10.1136/bmjopen-2024-095456 (PMC12121589; doi:10.1136/bmjopen-2024-095456)
Supplement: online supplemental file 1 [file bmjopen-15-5-s001.pdf]

Supplementary materials

Deviations from published protocol

Table S1 details the amendments made to the published protocol and the rationale.

Table S1: Details of amendments made to the published protocol

Details of amendment	Rationale
**Inclusion criteria: for intervention studies (RCTs and non-RCTs), only studies that have a significantly increased PAct score were included. Studies where the interventions had no effect on PAct scores or decreased it were excluded.**	The primary focus of this review is to assess the relationship between PAct and T2D-related outcomes. If an intervention did not increase PAct, then the observed change in T2D-related outcomes could be attributed to other mechanisms beyond the scope of this review. Excluding these studies eliminates potential confounding factors and strengthens the validity of the conclusions drawn.
**Included overall self-management score as an outcome**	There are several comprehensive self-management assessment tools for diabetes which yield an overall score that is a robust indicator of self-management[1–3]. This would also allow for a more comprehensive analysis and offers deeper insights into T2D-related outcomes in relation to PAct. For example, an increase in PAct scores might improve only one or two specific SMBs without affecting the overall self-management score due to its composite nature. Analysing these relationships would allow us to tailor interventions more precisely and identify key measures for clinical practice.
**We did not search the Health Management Information Consortium (HMIC) database, ZETOC and the British Library Integrated Catalogue for grey literature**	These sites were not searched due to resource limitations. However, for any posters or conference abstracts that seemed relevant, we contacted the authors for information about the study, and included it if the full-text was available.
**Only studies in English were included**	Unable to source for translation services due to limited resources
**Harvest plot design**	The design outlined in the protocol was modified to comprehensively capture and succinctly present the maximum amount of information available on the studies, including study ID, T2D-related outcomes analysed, sample size, quality, strength of design and hypothesised direction of association
**Levels of evidence were also reported for no or negative associations**	The approach to synthesise levels of evidence in the published protocol favours results in the positive direction.<br>Given that no or negative associations between PAct and T2D-related outcomes also have important implications for clinical practice, we decided to report the levels of evidence for *any* or *no* association based on consistency of the findings (similar to positive associations) to offer a more nuanced and balanced perspective of the heterogenous evidence base. This approach allows for a more precise evaluation of the associations and would enhance the clarity of evidence interpretation.

Search strategy

Table S2: Search strategy for Medline.

No	Search
**1**	("patient* activation*" or (measure* adj5 "patient activation") or PAM?22* or PAM?13* or PAM??13* or PAM??22* or "Patient Assessment of Chronic Illness Care*" or PACIC*).mp.
**2**	(Diabet* or T2DM or T1DM or (non insulin* depend* or non insulin depend* or non insulin?depend* or non insulin?depend* or IDDM or NIDDM or MODY or T1D or T2D)).mp. or exp Diabetes Mellitus, Type 2/ or exp Diabetes Mellitus/ or exp Diabetes Mellitus, Type 1/ or exp diabetes insipidus/ or exp Diabetes, Gestational/
**3**	1 and 2

Table S3: Search strategy for Embase.

No	Search
**1**	("patient* activation*" or (measure* adj5 "patient activation") or PAM?22* or PAM?13* or PAM??13* or PAM??22* or "Patient Assessment of Chronic Illness Care*" or PACIC*).mp.
**2**	(Diabet* or T2DM or T1DM or (non insulin* depend* or non insulin depend* or non insulin?depend* or non insulin?depend) or IDDM or NIDDM or MODY or T1D or T2DM).mp. or exp Diabetes Mellitus, Type 2/ or exp Diabetes Mellitus/ or exp Diabetes Mellitus, Type 1/ or exp diabetes insipidus/ or exp Diabetes, Gestational/
**3**	1 and 2

Table S4: Search strategy for CENTRAL.

No	Search
**#1**	(Patient* next activation*) or (measure* near/5 "patient activation") or PAM?22* or PAM?13* or PAM*13* or PAM*22*
**#2**	Diabet* or T2DM or T1DM or (non insulin* depend* or non insulin depend* or non insulin?depend* or non insulin?depend) or IDDM or NIDDM or T1D or T2D or [mh "Diabetes Mellitus, Type 2"] or [mh "Diabetes Mellitus"] or [mh "Diabetes Mellitus, Type 1"] or [mh "diabetes insipidus"] or [mh "Diabetes, Gestational"]
**#3**	1 and 2 in Trials

Table S5: Search strategy for PsycINFO.

No	Search
**S1**	("patient* activation*" or (measure* n5 "patient activation") or PAM?22* or PAM?13* or PAM??13* or PAM??22* or "Patient Assessment of Chronic Illness Care*" or PACIC*
**S2**	Diabet* or T2DM or T1DM or (non insulin* depend* or non insulin depend* or non insulin?depend* or non insulin?depend) or IDDM or NIDDM or MODY or T1D or T2D
**S3**	(DE "Diabetes Insipidus" OR DE "Diabetes Mellitus" OR DE "Diabetes" OR DE "Type 1 Diabetes" OR DE "Type 2 Diabetes") OR (DE "Gestational Diabetes")
**S4**	S2 OR S3
**S5**	S1 AND S4

Table S6: Search strategy for Web of Science.

No	Search
**1**	TS=("patient* activation*" or (measure* NEAR/5 "patient activation") or PAM?22* or PAM?13* or PAM??13* or PAM??22* or PAM$13 or PAM$22 or "Patient Assessment on Chronic Illness Care*" or PACIC*)
**2**	TS=(Diabet* or T2D or T1DM or (non insulin* depend* or non insulin depend* or non insulin?depend* or non insulin?depend) or IDDM or NIDDM or MODY or T1D or T2D or Diabetes or diabetes insipidus or gestational diabetes)
**3**	#1 and #2

Table S7: Search strategy for CINAHL.

No	Search
**S1**	"patient activation*" or (measure* N5 "patient activation") or PAM?22* or PAM?13* or PAM??13* or PAM??22* or PAM#13 or PAM#22 or "Patient Assessment on Chronic Illness Care*" or PACIC*
**S2**	Diabet* or T2DM or T1DM or (non insulin* depend* or non insulin depend* or non insulin?depend* or non insulin?depend) or IDDM or NIDDM or MODY or T1D or T2D
**S3**	(MH "Diabetes Mellitus, Type 2") OR (MH "Diabetes Mellitus, Type 1+") OR (MH "Diabetes Mellitus+") OR (MH "Diabetes Insipidus+") OR (MH "Diabetes Mellitus, Gestational")
**S4**	S2 OR S3
**S5**	S1 AND S4

Hypothesised association for each T2D-related outcome

Table S8: Direction of hypothesised association between higher PAct scores and T2D-related outcomes. The direction mentioned aligns with the preferable T2D-related outcome. For example, reduced HbA_{1c} levels are desirable, so higher PAct scores are hypothesised to correlate with lower HbA_{1c} levels, indicating a negative association.

Outcome	Hypothesised direction of association with higher PAct (i.e. association that corresponds to better T2D-related outcomes)
**Clinical outcomes**	
HbA_{1c} level	Negative [4–7]
Blood pressure	Negative [6–8]
Low-density lipoprotein (LDL)	Negative [6–8]
High-density lipoprotein (HDL)	Positive [7]
Total cholesterol	Negative[9]
Serum triglycerides	Negative[8]
Body mass index (BMI)	Negative[10]
Body weight	Negative[10]
**Self-management behaviours**	
Overall self-management score	Positive[9]
Diet	Positive[11–14]
Physical activity	Positive[11,15]
Smoking status	Negative[8,12]
Alcohol consumption	Negative[14]
Medication adherence	Positive[6,13,14]

Study design categorisation

Table S9: Categorisation of the suitability of various study designs (with corresponding analyses) to determine causal relationships between PAct and T2D-related outcomes. This table is reproduced from the protocol published by Mueller et al[16]. The table is distributed under the terms of the Creative Commons Attribution License (https://creativecommons.org/licenses/by/4.0/). No changes were made to the original table.

Possible study designs + analysis	Suitability of study design and analysis	Rationale
**RCTs with causal mediation analysis to assess whether PAct mediates intervention effects**	Strong	RCTs are the only study design that allow causal mediation analysis to identify the mechanisms by which interventions exert their effects[17]
**RCTs that do not report on the association between PAct and outcomes but that show intervention effects on outcomes AND intervention effects on PAct, AND the intervention explicitly, mainly addresses PAct**	Moderate	RCTs provide insight into causal effects of interventions on outcomes. If an intervention explicitly addresses PAct and there is evidence that the intervention influenced both PAct and outcomes, this provides indication for a causal mechanism of PAct on outcomes (though not definitive).
**Cohort studies/RCTs or other intervention studies that assess the association between PAct and subsequent outcomes**	Moderate	RCTs and longitudinal observational studies can provide temporal insights into the association between PAct and outcomes, which gives some indication of causality[18]. If an RCT examines the association between PAct and outcomes independent of study group allocation, randomisation has no bearing; analyses and findings are therefore akin to cohort studies.
**Observational cross-sectional studies**	Weak	In cross-sectional designs, the time order of effects cannot be determined and therefore causality cannot be inferred[19].
**Intervention studies that are not RCTs (eg, pre-post studies) and that do not report on the association between PAct and outcomes but that show changes in outcomes AND changes in PAct.**	Weak	Pre-post designs have the strength of temporality to indicate outcomes might be impacted by an intervention, but due to lack of randomisation causality cannot be inferred[20].

Levels of evidence synthesis

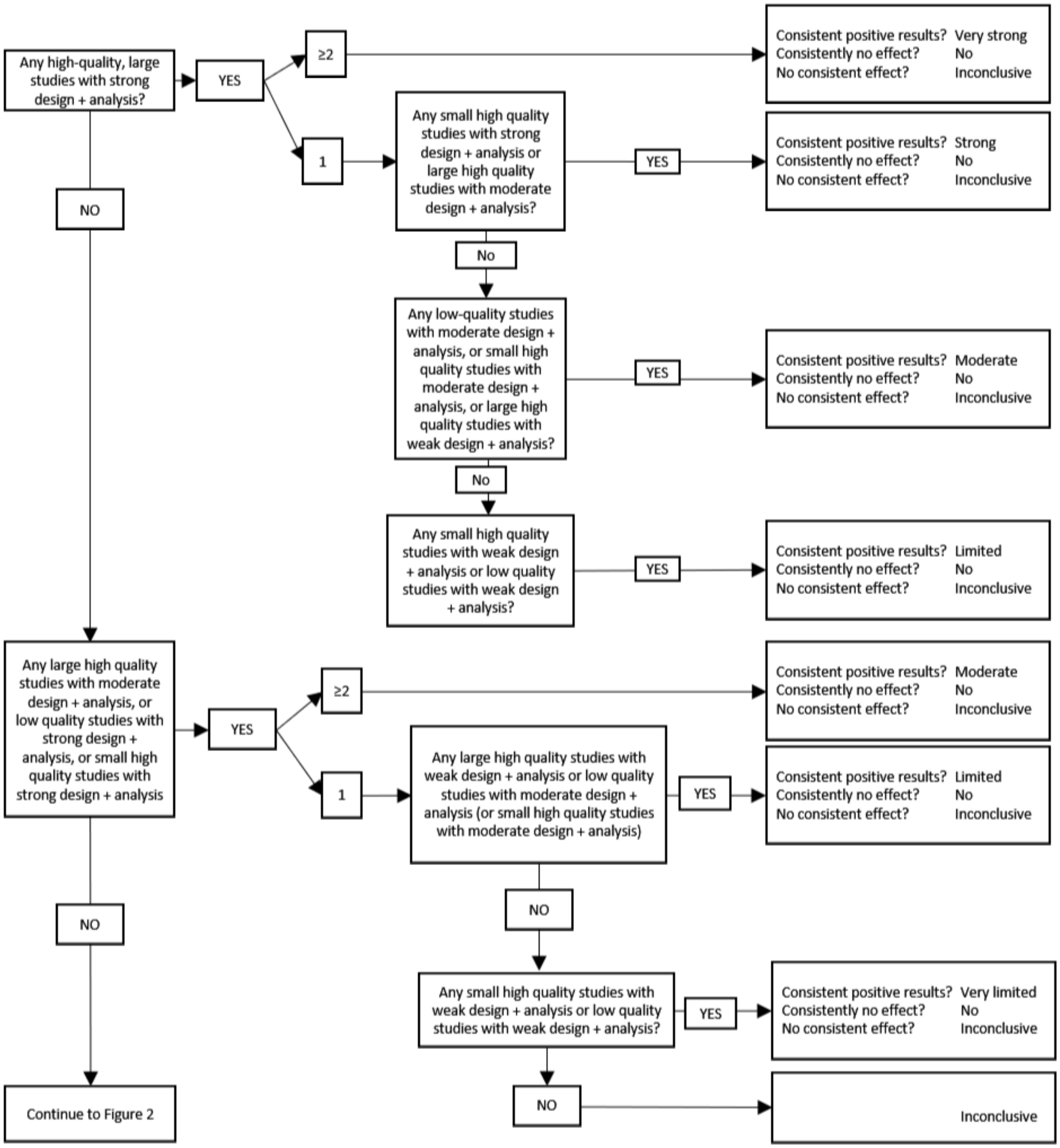

Figure S1: Levels of evidence PART 1 of 2. To be used together with Table S9 and Figure S2. Note: Studies with ≤250 participants or studies not providing a sample size justification are classified as 'small', while studies with >250 participants are classified as 'large'. Findings are deemed consistent if at least two-thirds (66.6%) of the highest quality studies report significant results in the same direction. This figure is reproduced from the protocol published by Mueller et al[16]. The figure is distributed under the terms of the Creative Commons Attribution License (https://creativecommons.org/licenses/by/4.0/). No changes were made to the original figure.

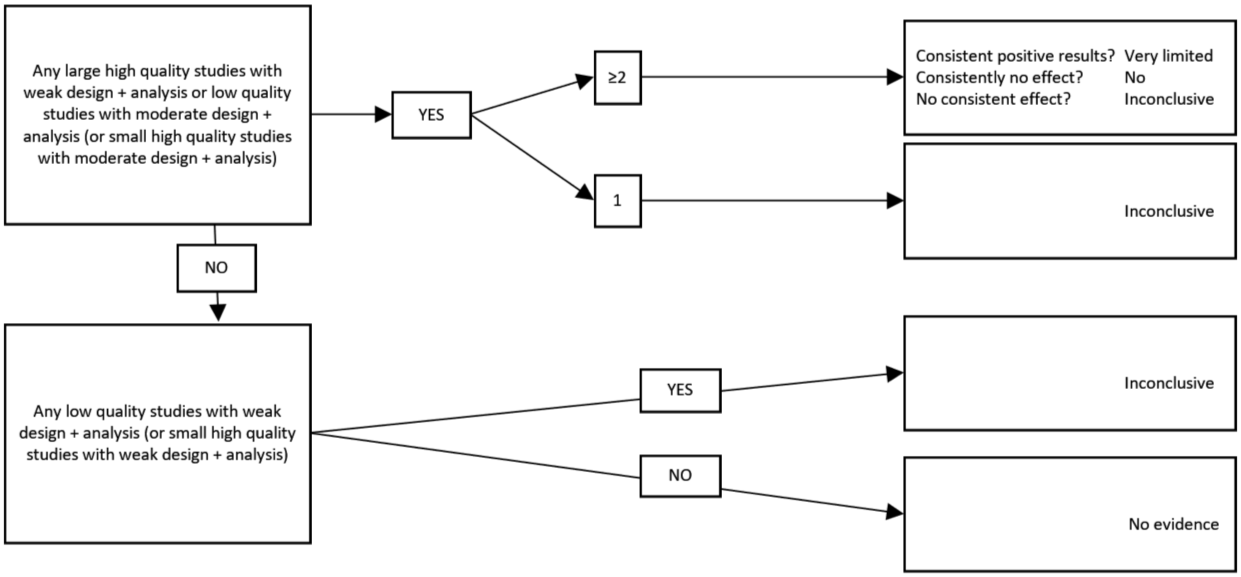

Figure S2: Levels of evidence PART 2 of 2. To be used together with Table S9 and Figure S1. Note: Studies with ≤250 participants or studies not providing a sample size justification are classified as 'small', while studies with >250 participants are classified as 'large'. Findings are deemed consistent if at least two-thirds (66.6%) of the highest quality studies report significant results in the same direction. This figure is reproduced from the protocol published by Mueller et al[16]. The figure is distributed under the terms of the Creative Commons Attribution License (https://creativecommons.org/licenses/by/4.0/). No changes were made to the original figure.

Risk of bias assessment for each study

Study	(Study ID) Author, year	Clear aim	≥50% of eligible persons participated	Loss to follow-up <20%	Sample size justified	Exposures measured prior to outcomes	Selection bias	Confounders adjusted for?	Blinding of outcome assessments	Incomplete outcome data	Selective outcome reporting	Overall risk
1	Almutairi 2023	Green	Yellow	Green	Yellow	Red	Red	Red	Yellow	Yellow	Green	Yellow
2	Almutairi 2023	Green	Yellow	N/A	Green	Red	Red	Yellow	Yellow	Yellow	Yellow	Yellow
3	Arvanitis 2020	Green	Yellow	N/A	Red	Red	Green	Red	Green	Yellow	Yellow	Yellow
4	Aung 2015	Green	Yellow	Green	Green	Green	Yellow	Red	Red	Green	Green	Red
5	Glenn 2020	Green	Red	N/A	Red	Red	Yellow	Red	Green	Yellow	Red	Red
6	Hendriks 2016	Green	Red	N/A	Green	Red	Green	Green	Green	Green	Green	Green
7	Kato 2020	Green	Yellow	N/A	Red	Red	Green	Green	Green	Green	Green	Green
8	Kim 2021	Green	Yellow	N/A	Red	Red	Red	Red	Yellow	Yellow	Yellow	Red
9	Ledford 2012	Green	Yellow	N/A	Red	Red	Green	Red	Yellow	Yellow	Yellow	Red

Study	(Study ID) Author, year	Clear aim	≥50% of eligible persons participated	Loss to follow-up <20%	Sample size justified	Exposures measured prior to outcomes	Selection bias	Confounders adjusted for?	Blinding of outcome assessments	Incomplete outcome data	Selective outcome reporting	Overall risk
10	Mayberry 2010	Green	Green	N/A	Red	Red	Yellow	Red	Yellow	Yellow	Green	Red
11	Michaud 2016	Green	Yellow	Red	Red	Green	Red	Green	Green	Yellow	Green	Yellow
12	Parchman 2010	Green	Yellow	N/A	Red	Red	Green	Green	Red	Green	Green	Green
13	Rask 2009	Green	Yellow	N/A	Red	Red	Red	Red	Yellow	Green	Green	Red
14	Regeer 2022	Green	Yellow	Green	Yellow	Green	Yellow	Green	Green	Yellow	Green	Green
15	Rogvi 2012	Green	Green	N/A	Red	Red	Green	Green	Green	Yellow	Green	Green
16	Shah 2015	Green	Yellow	Green	Green	Green	Green	Green	Green	Green	Yellow	Green
17	Stuart 2021	Green	Yellow	N/A	Red	Red	Yellow	Green	Green	Yellow	Green	Green
18	Su 2019	Green	Yellow	Red	Red	Green	Green	Green	Green	Yellow	Yellow	Green
19	Van Vugt 2018	Green	Yellow	N/A	Red	Red	Green	Green	Green	Green	Yellow	Green
20	Zhang 2023	Green	Yellow	N/A	Green	Red	Red	Red	Yellow	Yellow	Yellow	Red
21	Zheng 2019	Green	Yellow	N/A	Red	Red	Green	Green	Yellow	Yellow	Green	Green

Figure S3: Risk of bias assessment for longitudinal, pre-post intervention and cross-sectional studies on the top diagram. No RCTs met the inclusion criteria. Loss to follow-up N/A for all cross-sectional studies.

References:

1. Schmitt A, Kulzer B, Ehrmann D, Haak T, Hermanns N. A Self-Report Measure of Diabetes Self-Management for Type 1 and Type 2 Diabetes: The Diabetes Self-Management Questionnaire-Revised (DSMQ-R) – Clinimetric Evidence From Five Studies. *Front Clin Diabetes Healthc*. 2022;2:823046. doi:10.3389/fcdhc.2021.823046

2. Peyrot M, Bushnell DM, Best JH, Martin ML, Cameron A, Patrick DL. Development and validation of the self-management profile for type 2 diabetes (SMP-T2D). *Health Qual Life Outcomes*. 2012;10(1):125. doi:10.1186/1477-7525-10-125

3. Toobert DJ, Hampson SE, Glasgow RE. The summary of diabetes self-care activities measure: results from 7 studies and a revised scale. *Diabetes Care*. 2000;23(7):943-950. doi:10.2337/diacare.23.7.943

4. Remmers C, Hibbard J, Mosen DM, Wagenfield M, Hoye RE, Jones C. Is Patient Activation Associated With Future Health Outcomes and Healthcare Utilization Among Patients With Diabetes? *J Ambulatory Care Manage*. 2009;32(4):320-327. doi:10.1097/JAC.0b013e3181ba6e77

5. Rogvi S, Tapager I, Almdal TP, Schiøtz ML, Willaing I. Patient factors and glycaemic control – associations and explanatory power. *Diabet Med*. 2012;29(10). doi:10.1111/j.1464-5491.2012.03703.x

6. Parchman ML, Zeber JE, Palmer RF. Participatory Decision Making, Patient Activation, Medication Adherence, and Intermediate Clinical Outcomes in Type 2 Diabetes: A STARNet Study. *Ann Fam Med*. 2010;8(5):410-417. doi:10.1370/afm.1161

7. Sacks RM, Greene J, Hibbard J, Overton V, Parrotta CD. Does patient activation predict the course of type 2 diabetes? A longitudinal study. *Patient Educ Couns*. 2017;100(7):1268-1275. doi:10.1016/j.pec.2017.01.014

8. Greene J, Hibbard JH. Why Does Patient Activation Matter? An Examination of the Relationships Between Patient Activation and Health-Related Outcomes. *J Gen Intern Med*. 2012;27(5):520-526. doi:10.1007/s11606-011-1931-2

9. Almutairi N, Gopaldasani V, Hosseinzadeh H. The Effect of a Patient Activation Tailored Intervention on Type 2 Diabetes Self-Management and Clinical Outcomes: A Study from Saudi Arabian Primary Care Settings. *J Diabetes Res*. 2023;2023:2074560. doi:10.1155/2023/2074560

10. Regeer H, van Empelen P, Bilo HJG, de Koning EJP, Huisman SD. Change is possible: How increased patient activation is associated with favorable changes in well-being, self-management and health outcomes among people with type 2 diabetes mellitus: A prospective longitudinal study. *Patient Educ Couns*. 2022;105(4):821-827. doi:10.1016/j.pec.2021.07.014

11. Almutairi N, Hosseinzadeh H, Gopaldasani V. The effectiveness of patient activation intervention on type 2 diabetes mellitus glycemic control and self-management behaviors: A systematic review of RCTs. *Prim Care Diabetes*. 2020;14(1):12-20. doi:10.1016/j.pcd.2019.08.009

12. Hibbard JH, Stockard J, Mahoney ER, Tusler M. Development of the Patient Activation Measure (PAM): Conceptualizing and Measuring Activation in Patients and Consumers. *Health Serv Res*. 2004;39(4p1):1005-1026. doi:10.1111/j.1475-6773.2004.00269.x

13. Hibbard JH, Mahoney ER, Stock R, Tusler M. Do Increases in Patient Activation Result in Improved Self-Management Behaviors? *Health Serv Res*. 2007;42(4):1443-1463. doi:10.1111/j.1475-6773.2006.00669.x

14. Paukkonen L, Oikarinen A, Kähkönen O, Kaakinen P. Patient activation for self-management among adult patients with multimorbidity in primary healthcare settings. *Health Sci Rep*. 2022;5(4):e735. doi:10.1002/hsr2.735

15. Rask KJ, Ziemer DC, Kohler SA, Hawley JN, Arinde FJ, Barnes CS. Patient Activation Is Associated With Healthy Behaviors and Ease in Managing Diabetes in an Indigent Population. *Diabetes Educ*. 2009;35(4):622-630. doi:10.1177/0145721709335004

16. Mueller J, Ahern AL, Sharp SJ, et al. Association between patient activation, self-management behaviours and clinical outcomes in adults with diabetes or related metabolic disorders: a systematic review and meta-analysis protocol. *BMJ Open*. 2022;12(1):e056293. doi:10.1136/bmjopen-2021-056293

17. Lee H, Herbert RD, Lamb SE, Moseley AM, McAuley JH. Investigating causal mechanisms in randomised controlled trials. *Trials*. 2019;20(1):1-5. doi:10.1186/s13063-019-3593-z

18. Barnett ML, Hyman JJ. Challenges in interpreting study results: the conflict between appearance and reality. *J Am Dent Assoc 1939*. 2006;137 Suppl:32S-36S. doi:10.14219/jada.archive.2006.0405

19. Porta MS, Greenland S, Hernán M, Silva I dos S, Last JM, International Epidemiological Association, eds. *A Dictionary of Epidemiology*. 6th edition. Oxford University Press; 2014.

20. Thiese MS. Observational and interventional study design types; an overview. *Biochem Medica*. 2014;24(2):199-210. doi:10.11613/BM.2014.022
