# Supplementary material for: Association between patient activation, self-management behaviours and clinical outcomes in adults with type 2 diabetes: a systematic review with narrative synthesis
Source: BMJ Open. 2025 May 27;15(5):e095456. doi: 10.1136/bmjopen-2024-095456 (PMC12121589; doi:10.1136/bmjopen-2024-095456)
Supplement: online supplemental file 2 [file bmjopen-15-5-s002.docx]

# Data extraction sheet

#### Section 1: General meta-data

| **Review title** | The association between patient activation, self-management behaviours and clinical outcomes in adults with diabetes or related metabolic disorders: A systematic review and meta-analysis |
| --- | --- |
| **Study ID** *(surname of first author and year first full report of study was published e.g. Smith 2001)* |  |
| **Date form completed** *(dd/mm/yyyy)* |  |
| **Initials of person extracting data:** |  |
| **Title:** |  |
| **Source (e.g. name of journal):** |  |
| **Publication type** (e.g. article, dissertation) |  |

#### Section 2: Objectives and design

| **Objective:** |  |
| --- | --- |
| **Setting (e.g. community setting, primary care etc.):** |  |
| **Country of origin:** |  |
| **Study design:** | **Actual**:  **For this review**:  *Please note the actual study design AND the one for the purpose of this paper, e.g. if it is an RCT but we are extracting information on the relationship between activation and outcomes regardless of study group, then we are treating it as a cohort study* |
| **Study population:** |  |
| **Recruitment methods:** |  |
| **Inclusion and exclusion criteria for participants:** |  |
| **Sample size:** |  |
| **Is a justification for the sample size provided (power calculation)?** | Yes/No (delete as appropriate)  Details: |
| **Withdrawals and exclusions:** |  |
| **Attrition (i.e. loss to follow-up, %):**  **(For intervention studies, report per study group)** |  |

#### Section 3: Intervention details

**Only complete Section 3 if it is an intervention study and we are interested in findings that depend on study group allocation.** If it is an observational study, or an intervention study but the relevant data to extract pertain to the association between PA and outcomes independent of study group allocation, skip to section 4.

|  | **Descriptions as stated in the report/paper** |
| --- | --- |
| **Randomisation and blinding:** |  |
| **Sample size per group** | Intervention:  Control: |
| **Any indication for baseline differences between study groups?** | Yes/No/Unclear  Details: |
| **Comparison group description** |  |
| **Intervention aim** |  |
| **Is the explicit main aim of the intervention to increase patient activation or to target patients’ knowledge, confidence and skills for self-management?** | *Yes/No/Unclear*  *(Delete as appropriate.*  *Select No if the patient activation component forms part of a larger complex intervention).* |
| **Is patient activation the main component of the intervention?** |  |
| **Intervention description** |  |
| **Group or individual delivery** |  |
| **Mode of delivery (e.g. web, face-to-face)** |  |
| **Duration of intervention** |  |
| **Timing (e.g. frequency, duration of each session)** |  |
| **Providers (e.g. profession and training received)** |  |
| **Intention to treat analysis?** | *Yes/No/Unclear (Delete as appropriate).* |
| **Any further notes:** |  |

#### Section 4: Outcomes & Measures

| **Patient Activation (PA) measure** |  |
| --- | --- |
| **PA measure used as continuous measure, ordinal (levels 1-4), or dichotomous (high/low e.g. levels1/2 vs. levels 3/4)?** | Continuous/ordinal/dichotomous  (delete as appropriate) |
| **Time points measured/reported (for all outcomes):** |  |

##### Clinical outcomes

*Note: If outcomes not measured, please insert “n/a”*

|  | **How measured/defined (+unit of measurement and any cut-offs used)** | **Source (e.g. self-report, medical records)** |
| --- | --- | --- |
| HbA1C level/glycaemic control |  |  |
| Systolic blood pressure, diastolic blood pressure |  |  |
| Low-density lipoprotein (LDL)  High-density lipoprotein (HDL)  Total cholesterol |  |  |
| Serum triglycerides |  |  |
| BMI |  |  |
| weight |  |  |

##### Self-management behaviours

*Note: If outcomes not measured, please insert “n/a”*

|  | **Self-report? (Yes/No/Unclear)** | **How defined/measured? *e.g. “consuming 5 servings of fruit/veg per day (Yes/No)”*** |
| --- | --- | --- |
| Diet |  |  |
| Physical activity |  |  |
| Smoking |  |  |
| Alcohol consumption |  |  |
| Medication adherence |  |  |

#### Section 5: Analyses + Results

*Please extract data for adjusted and unadjusted associations where both are reported (i.e. associations just between PA and the relevant outcome [=unadjusted], and those where other confounders are added to the model to control for confounders [=adjusted]).*

*If several time points are reported, extract data for the longest follow-up time point.*

*If several variables were used for the same outcome please copy and paste the table and add details for the respective variable (for example, create a second table for “diet”, and add the variable).*

*If the format of the tables is unsuitable for the reported results, please paste the relevant results into the ‘other/comments’ section.*

| How were missing data handled? *(e.g. multiple imputation)* |  |
| --- | --- |

| **Outcome: HbA1c/glycaemic control** |  |
| --- | --- |
| **Type of association:**  *(delete as appropriate)* | - Cross-sectional association of outcomes with PA - longitudinal: Association between baseline PA and subsequent outcome - longitudinal: Association between baseline PA and change in outcome - longitudinal: Association between change in PA and subsequent outcome - longitudinal: Association between change in PA and change in outcome |
| **If longitudinal: Length of time between the two measurement timepoints** |  |
| **Statistical test (e.g. correlation r, t-test, linear regression, logistic regression…)** |  |
| **Relevant statistical parameters** (e.g. correlation r, χ2, F, Odds ratios, beta coefficients, with p-values) **[unadjusted]** |  |
| **Relevant statistical parameters** (e.g. correlation r, χ2, F, Odds ratios, beta coefficients, with p-values) **[adjusted]** | **Covariates:** |
| **Sample size:** |  |
| **Other/comments:** |  |

*To extract data for further outcomes, please copy and paste the table above and edit the “outcome” field.*

Outcomes:

- systolic blood pressure
- diastolic blood pressure
- LDL/HDL/Total cholesterol
- serum triglycerides
- weight
- BMI
- Diet
- Physical activity
- Smoking
- Alcohol
- Medication adherence

#### Section 6: Conclusions

*Please note any comments here.*

| **Reviewer’s conclusions/comments:** |  |
| --- | --- |
