# Supplementary material for: Association between patient activation, self-management behaviours and clinical outcomes in adults with type 2 diabetes: a systematic review with narrative synthesis
Source: BMJ Open. 2025 May 27;15(5):e095456. doi: 10.1136/bmjopen-2024-095456 (PMC12121589; doi:10.1136/bmjopen-2024-095456)
Supplement: Supplementary file 3 [file bmjopen-2024-095456.reviewer_comments.pdf]

PEER REVIEW HISTORY

BMJ Open publishes all reviews undertaken for accepted manuscripts. Reviewers are asked to complete a checklist review form and are provided with free text boxes to elaborate on their assessment. These free text comments are reproduced below.

ARTICLE DETAILS

Title (Provisional)

Association between patient activation, self-management behaviours, and clinical outcomes in adults with type 2 diabetes: a systematic review with narrative synthesis

Authors

Thinakaran, Koghanadhacharve; Ahern, Amy; Beckett, Robert S; Shaida, Sara F; Wills, Harriet M; Richards , Rebecca; Birch, Jack M; Griffin, Simon J; Mueller, Julia

---

VERSION 1 - REVIEW

**Reviewer**	**1**
**Name**	**Freeby, Matthew**
**Affiliation**	**University of California Los Angeles David Geffen School of Medicine**
**Date**	**30-Nov-2024**
**COI**	**None**

In this systematic review with narrative synthesis, the authors provide a comprehensive summary of the evidence for patient activation in type 2 diabetes mellitus. In 2022, they provided a systematic review and meta-analysis protocol. The methods, outcomes, conclusion and limitations are well-described. The following edits should be considered:

1) Although PAct is more clearly described in the first review, it would be useful to provide a brief explanation of PAM, IMPACT-D, Lorig Communications Scale in the second review.

2) In 6.4 Quality Appraisal, please describe reasoning for low quality studies

3) Table 3 should be more succinctly summarized. It is longer than needed and can be better highlighted.

**Reviewer**	**2**
**Name**	**Dintsios, Charalabos-Markos**

**Affiliation**	**Heinrich Heine University Düsseldorf, Medical Faculty**
**Date**	**04-Feb-2025**
**COI**	**None**

A very interesting systematic review on a topic of high importance. I have only a few comments, which hopefully can ameliorate the manuscript.

P2L33-40: I would suggest to include in the keywords the term 'patient activation' as well or in case you have exceed the number to replace 'patient participation' with the above mentioned term.

P7L6-10 (Chapter 5.4): Please include at least in the supplement the data extraction sheet.

P8L14-18: Please elaborate a bit more on the adapted 'level of evidence' composite assessment, since it would be easier for the readership to be informed on the adaptation than trying to find out himself.

P14L17 (Discussion): Maybe the 'and' in the sentence referring to the two other RCTs should be replaced by an 'or'.

The resolution of the figures S1 and S2 is really bad. Pls. try to increase the resolution, if possible.

VERSION 1 - AUTHOR RESPONSE

Reviewer: 1
Dr. Matthew Freeby, University of California Los Angeles David Geffen School of Medicine
Comments to the Author:
In this systematic review with narrative synthesis, the authors provide a comprehensive summary of the evidence for patient activation in type 2 diabetes mellitus. In 2022, they provided a systematic review and meta-analysis protocol. The methods, outcomes, conclusion and limitations are well-described. The following edits should be considered:

1. Although PAct is more clearly described in the first review, it would be useful to provide a brief explanation of PAM, IMPACT-D, Lorig Communications Scale in the second review.
   We agree that a brief mention of some tools is necessary. As such we have included this sentence in the introduction:

   PAct is measured using various tools, like the Patient Activation Measure (PAM)(4), which is the most prevalent method and also used within the UK NHS. Other general instruments like the Patient Assessment of Chronic Illness Care (PACIC) (5) and T2D-specific tools like the Influence and Motivation for Patient

ACTivation in Diabetes care (IMPACT-D™)(6) are also employed in certain settings.

2. In 6.4 Quality Appraisal, please describe reasoning for low quality studies

   We have addressed this in an addition to section 6.4:
   Most studies were low-quality due to selection bias and failing to adjust for potential confounders. Particularly in the context of this review, factors such as age, disease duration and comorbidities can influence both PAct and outcomes, so should be accounted for in the analyses. Furthermore, the reliance on self-reported questionnaires to measure PAct predisposes the studies to selection bias, which is exacerbated by convenience sampling or the selective inclusion of patients with digital tools, potentially limiting generalisability.

3. Table 3 should be more succinctly summarized. It is longer than needed and can be better highlighted.

   We have made edits to reduce the number of words in Table 3 and to improve the readability.

Reviewer: 2
Dr. Charalabos-Markos Dintsios, Heinrich Heine University Düsseldorf
Comments to the Author:
A very interesting systematic review on a topic of high importance. I have only a few comments, which hopefully can ameliorate the manuscript.

1. P2L33-40: I would suggest to include in the keywords the term 'patient activation' as well or in case you have exceed the number to replace 'patient participation' with the above mentioned term.
   This an excellent suggestion, but the automatic drop-down box on the submission site did not have ‘patient activation’, therefore we went with the next best alternative.

2. P7L6-10 (Chapter 5.4): Please include at least in the supplement the data extraction sheet.
   This has been included.

3. P8L14-18: Please elaborate a bit more on the adapted 'level of evidence' composite assessment, since it would be easier for the readership to be informed on the adaptation than trying to find out himself.
   The last paragraph in Section 5.6 now reads:

The ‘level of evidence’ is a composite assessment synthesised from the strength of the study design/ analysis, the study quality, sample size and consistency of the findings, adapted from an approach used in a prior systematic review(40) **to include the strength of the study design/ analysis and the sample size**.

4. P14L17 (Discussion): Maybe the 'and' in the sentence referring to the two other RCTs should be replaced by an 'or'.
   This has been amended.
5. The resolution of the figures S1 and S2 is really bad. Pls. try to increase the resolution, if possible.
   That could possibly have happened in the process of inserting the pictures in a Word document. We have amended them and hope they appear clearer, but are also happy to provide PowerPoint/ JPEG images of figures S1 and S2 if that would help.
